# Official List of Changes of Stations and Duties of Medical Officers of the United States Marine Hospital Service

**Published:** 1884-09

**Authors:** 


### MARINE HOSPITAL SERVICE.

OFFICIAL LIST OF CHANGES OF STATIONS AND DUTIES OF MEDICAL OFFICERS OF THE UNITED STATES MARINE HOSPITAL SERVICE. *April 1st to June 30, 1884.*

Bailhache, P. H., Surgeon. Detailed as chairman of Board to examine candidate for appointment into the Revenue Marine Service, May 17, 1884.

Vansant, John, Surgeon. To proceed to Empire City, Oregon, as inspector, April 2, 1884.

Hutton, W. H. H., Surgeon. Granted leave of absence for twenty-five days, May 14 and June 9, 1884.

Miller, T. W., Surgeon. Granted leave of absence to attend the meeting of the American Medical Association, May 1, 1884. To proceed to Pittsburgh, Pa., Ashtabula, Ohio, Buffalo, N. Y., and Detroit, Mich., as inspector, May 10, 1884.

Wyman, Walter, Surgeon. To proceed to Crisfield, Md., as inspector, April 11, 1884. Detailed as president of Board for physical examination of candidates for appointment as cadets in the Revenue Marine Service, May 20, 1884. To examine cadet graduates Revenue Marine Service as to physical qualifications, May 31, 1884.

Detailed as member of Commission to inspect United States buildings at quarantine station on the Delaware River, June 16, 1884.

Wyman, Walter, Surgeon. Detailed to represent the Marine Hospital Service as delegate to the American Medical Association, April 17, 1884.

Austin, H. W., Surgeon. Granted leave of absence to attend the meeting of the American Medical Association, May 2, 1884.

Gassaway, J. M., Surgeon. When relieved by P. A. Surgeon Mead to proceed to Portland, Maine, and assume charge of the Service, April 16, 1884.

Granted leave of absence for thirty days, May 28, 1884.

Stoner, G. W., Passed Ass't Surgeon. When relieved by Surgeon Gassaway to proceed to Cairo, Ill., and assume charge of the Service, April 16, 1884.

When relieved by Surgeon Gassaway, to report in person to the Surgeon-General, June 20, 1884.

Irwin, Fairfax, Passed Ass't Surgeon. Granted leave of absence for twenty-one days, June 19, 1884.

Mead, F. W., Passed Ass't Surgeon. When relieved by Ass't Surgeon Devan, to proceed to Philadelphia, Pa., and assume charge of the Service, April 16, 1884.

Detailed as recorder of Board for physical examination of candidates for appointment as cadets in the Revenue Marine Service, May 20, 1884.

Carter, H. R., Passed Ass't Surgeon. To inspect unserviceable property at the San Francisco Hospital, May 24, 1884.

Wheeler, W. A., Passed Ass't Surgeon. To inspect unserviceable property at the Chicago Hospital, May 24, 1884.

Benson, J. A., Passed Ass't Surgeon. Granted leave of absence for thirty days, April 14, 1884.

When relieved by P. A. Surgeon Stone, to report to him for temporary duty, May 19, 1884.

Banks, C. E., Passed Ass't Surgeon. Detai led as member of

Board to examine physically candidate for appointment into the Revenue Marine Service, May 17, 1884.

To inspect unserviceable property at Baltimore, Md., New York, N. Y., and Boston, Mass., May 26 and June 2, 1884.

Bennett, P. H., Ass't Surgeon. Granted leave of absence for twenty days, June 28, 1884.

Devan, S. C., Ass't Surgeon. To proceed to Port Townsend, W. T., relieve P. A. Surgeon Mead, and assume charge of the Service, April 14, 1884.

Urquhart, F. M., Ass't Surgeon. Granted leave of absence for thirty days, May 22, 1884.

Yemans, H. W., Ass't Surgeon. To report to Capt. M. A. Healey for duty as medical officer during cruise of Revenue Cutter "Corwin," April 16, 1884.

Glennan, A. H., Ass't Surgeon. To proceed to Mobile, Ala., for temporary duty during sickness of P. A. Surgeon Goldborough, June 17, 1884.

APPOINTMENT.—Brooks, Stephen D., M. D., of Massachusetts, having passed the examination required by the Regulations, was appointed as Assistant Surgeon by the Secretary of the Treasury, May 15, 1884.

(Dr. Brooks had previously served as an Acting Assistant Surgeon, from March, 1883, to May, 1884.)

